# Mechanically Alloyed CoCrFeNiMo_0.85_ High-Entropy Alloy for Corrosion Resistance Coatings

**DOI:** 10.3390/ma14143802

**Published:** 2021-07-07

**Authors:** Laura Elena Geambazu, Cosmin Mihai Cotruţ, Florin Miculescu, Ioana Csaki

**Affiliations:** Material Science and Engineering Faculty, University Politehnica of Bucharest, 060042 Bucuresti, Romania; laura.geambazu@gmail.com (L.E.G.); florin.miculescu@gmail.com (F.M.); ioana.apostolescu@upb.ro (I.C.)

**Keywords:** high-entropy alloys, mechanical alloying, coatings, adhesion

## Abstract

High-entropy alloys could provide a solution for corrosion resistance due to their impressive properties. Solid-state processing of high purity Co, Cr, Fe, Ni and Mo metallic powders and consolidation resulted in a bulk material that was further machined into electro spark deposition electrodes. After the stainless steel substrate surface preparation, thin successive layers of the high-entropy alloy were deposited and Pull-Off testing was performed on the newly obtained coating, for a better understanding of the adhesion efficiency of this technique. Good adhesion of the coating to the substrate was proved by the test and no cracks or exfoliations were present. Corrosion resistance testing was performed in a liquid solution of 3.5 wt.% NaCl for 6 h at room temperature and the results obtained validated our hypothesis that CoCrFeNiMo_0.85_ high-entropy alloys could provide corrosion resistance when coating a stainless steel substrate.

## 1. Introduction

The topic of high-entropy alloys was investigated by a couple of studies during the past decade. The possibility of composition designing according to a targeted application was an appealing property of these types of alloys [1,2,3]. The studies were conducted on different compositions, mechanical properties, thermal resistance and microstructures [4,5,6].

The corrosion and erosion resistance of CoCrFeNiMo_0.85_ coatings manufactured by laser metal deposition technique was previously tested in a geothermal environment [7]. The results revealed good erosion resistance and no corrosion damage when exposed to different conditions, leading to the conclusion that this type of newly developed coating is suitable for aggressive media

Zihui Dong et al. [8] studied CoCrFeMo_0/85_Ni powder, produced by atomization, for differential thermal analyses and differential scanning calorimetry to assess the thermodynamic properties including solidus, liquidus and phase transitions temperatures. Their study revealed that the complex FCC matrix of the high-entropy alloy was embedded with multiple phases which are the mixture of the Co-, Cr-, Fe-rich tetragonal phase and Mo-rich rhombohedral phases. The mixture of these phases could enhance the HEA properties, including corrosion resistance.

Corrosion resistance-related issues are often reported in the renewable energy industry [9,10,11,12,13] where the operating efficiency of the equipment and the maintenance costs are affected by it.

Our goal was to design high corrosion resistance coatings for components in the geothermal industry. Due to the chemical composition of the geothermal steam, high temperatures, high pressure and abrasive elements such as sand result in a highly aggressive medium for the in-work equipment [14].

Almar Gunnarsson et al. [15] reported the corrosion–erosion and wear effect on the turbine blades operating in a geothermal environment. Cost reduction in maintenance is required in order to increase the competitiveness of this type of resource in the renewable energy market. A solution might be represented by developing corrosive resistant coatings that can be deposited on the turbine blades, but also local repairs.

The high-entropy alloys, known for their improved performances and properties are studied [16,17,18,19] and represent a focal point in modern-day industrial technology. The specific effects exhibited and the versatility of this type of materials resulted in studies on the processing methods and designed properties that can be obtained for specific end users.

One of our first studies [20] was focused on the in situ corrosion resistance of CoCrFeNiMo bulk high-entropy alloy obtained by vacuum arc remelting, tested in a geothermal power plant. The results indicated a mass loss of ~0.002%, correlated with a corrosion rate of 0.00034 mm/year, when the samples were tested in direct contact with the geothermal steam for a period of 30 days.

The aim of this paper was to research corrosion behavior of novel coatings, produced by electro spark deposition, for the geothermal industry. The coatings were produced using original electrodes, produced in-house from CoCrFeNiMo high-entropy alloy bulk material. The electrodes for electro spark deposition were produced by mechanical alloying, pressing and sintering. Mechanical alloying is a technique for HEA production that ensures a higher homogenization and alloying degree. The mechanically alloyed bulk samples were consolidated and machined into electrodes designed for the electro spark deposition equipment and new high-entropy coatings were developed. Our report presents the microstructure and corrosions resistance results on this original coating.

## 2. Materials and Methods

The goal of the paper was to investigate the properties of the CoCrFeNiMo_0.85_ high-entropy alloy produced by solid-state processing and deposited on 316 L stainless steel substrates by electro spark deposition technique under argon atmosphere.

The alloy was prepared by powder metallurgy processing route. High purity, raw metallic powders of cobalt, chromium, iron, nickel and molybdenum were mechanically alloyed for 30 h in a Pulverisette 6 Classic Line planetary ball mill (Fritsch®, Idar-Oberstein, Germany), with stainless steel balls and vial. To ensure a good milling efficiency, wet milling was considered using N-Heptane as a process control agent. Other milling parameters consist of ball to powder ratio of 10:1 and 350 RPM. For the entire process, powder handling and processing were performed under Argon atmosphere in order to reduce the oxygen contamination in the final alloy.

The alloyed metallic powder was consolidated by cold pressing in Walter+Baiag LFV-300 kN equipmen, Löhningen, Switzerland with a 250 kN pressing force, into dia. 25 mm × 5 mm samples. The samples dimension was decided accordingly with the desired final dimensions of the electrode.

After sintering at 1100 °C for 1 h, the samples were machined into electrodes that were further used to coat stainless steel surfaces by electro spark deposition technique. In order to deposit high-entropy alloy layers, Spark Depo Model 3 with a miniature applicator was used and the deposition parameters are presented in Table 1.

**Figure 1:**
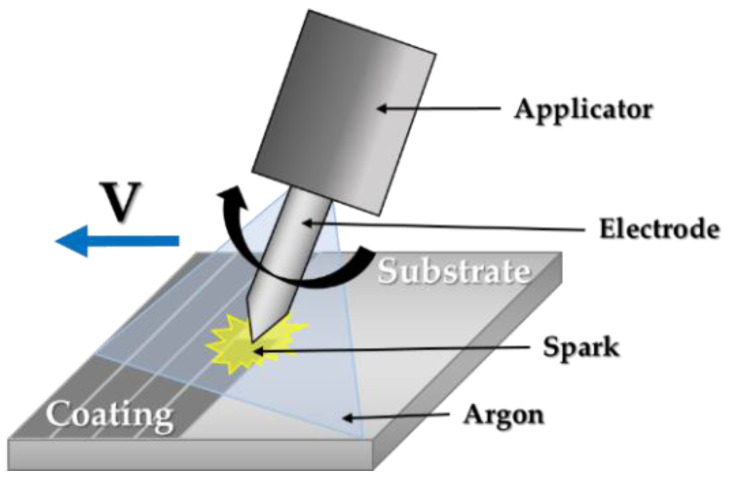
Graphic representation of the electro spark deposition technique.

Successive layers were deposited under argon atmosphere as illustrated in Figure 1. According to the literature [21,22,23,24], the desired coating thickness is between 25 µm and 100 µm. For a thickness less than 25 µm there are no major improvements and for over 100 µm thickness, possible embrittlement was reported.

After each producing step of the electrode, the microstructure of the samples was investigated. The microstructure of the coating was investigated from the top view and cross-section. The cross-section of the ESD specimen was mounted in thermosetting phenol formaldehyde resin (Bakelite). The specimens were ground and polished with SiC abrasive paper (down to 1200 grit) for the SEM and EDS analysis. A scanning electron microscope FEI Philips with an EDAX Saphire electron dispersive energy was used to analyze the specimens.

In order to test the adhesion of the deposited material, Pull-Off testing was performed according to ASTM D4541–“Standard Test Method for Pull-Off Strength of Coatings Using Portable Adhesion Testers” [25] and the surfaces were prepared by ASTM D2651-01(2016) “–Standard Guide for Preparation of Metal Surfaces for Adhesive Bonding” [26]. For the experiments, a pull-off testing device was crafted out of stainless steel. Bi-component epoxy resin was used as adhesive glue between the sample and the pulling rod, with an adhesive resistance of approx. 21 MPa according to the manufacturer. For maximum performance, the epoxy resin was applied 24 h prior to testing and the results are presented.

The corrosion resistance of the coated surfaces was tested by using the Galvanostat/Potentiostat model PARSTAT 4000, equipment (Princeton Applied Research–Ametek, Oak Ridge, TN, USA) in an electrochemical cell at the temperature of 25 ± 0.5 °C in a saline solution of 3.5 wt.% NaCl for 6 h.

## 3. Results

### 3.1. Microstructure Investigation for CoCrFeNiMo_0.85_ High-Entropy Alloy Coatings

**Figure 2:**
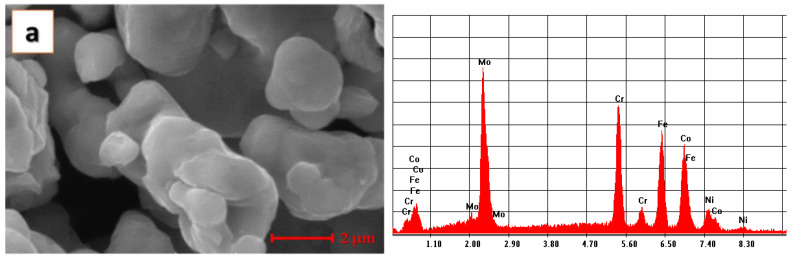

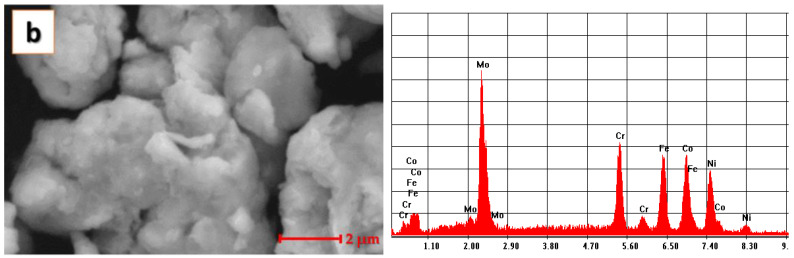
SEM images and results from EDS analyses for the powders used for CoCrFeNiMo_0.85_ HEA producing (**a**) homogenized powder, (**b**) mechanically alloyed powder.

The high-entropy alloy CoCrFeNiMo_0.85_ was processed by mechanical alloying. The microstructures of the initial and alloyed powders are presented in Figure 2.

In Figure 2a, the results of the microstructural analyses present the homogenized powder and the chemical composition confirmed by the results of the EDS analyses. In Figure 2b, the alloyed HEA can be observed after 30 h of milling. The results of the SEM and EDS analyses present a high alloying degree and a good homogenization, with no contamination or oxygen in the chemical composition.

**Figure 3:**
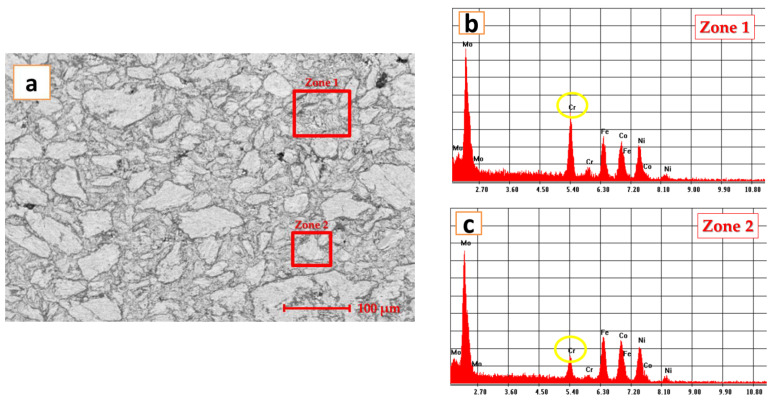
Analyses results of CoCrFeNiMo_0.85_ HEA pressed and sintered (**a**) SEM image of the sintered sample, (**b**) EDS analyses for zone 1 and (**c**) EDS analyses for zone 2.

The produced powders were pressed and sintered as was reported previously [27]. The SEM images of the sintered electrode are presented in Figure 3.

The EDS analyses were performed on the bulk solid solution and on the light grey phases present as cuboids particles in the solid solution. The cuboids are rich in Mo and present fewer Cr particles in comparison with the bulk solid solution richer in Cr.

**Figure 4:**
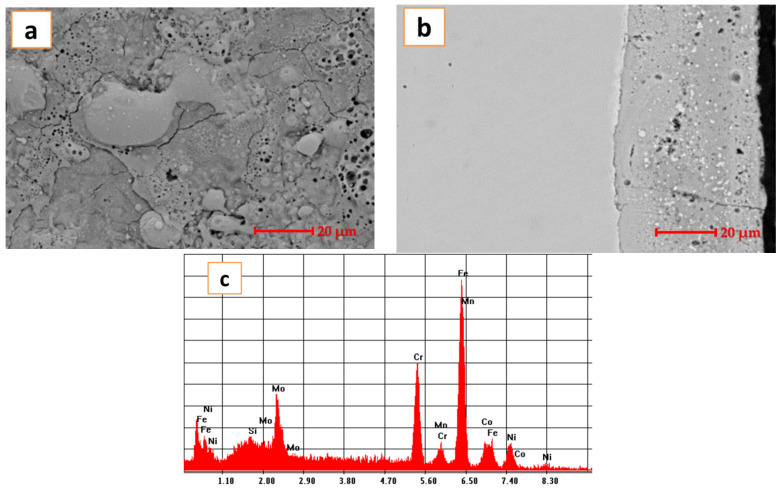
CoCrFeNiMo_0.85_ HEA coating; (**a**) SEM images of the top view (**b**) SEM images of the cross-section (**c**) EDS analyses results for the coating.

The coating obtained with the processed electrodes is presented in Figure 4.

The top view of the coating reveals some pores and some splats on the surface. The cross-section confirms a slightly porous coating in the upper part but adhesive and homogeneous. The results of the EDS analyses reveal a slight interference of the substrate with the coated layer.

### 3.2. XRD of the Sintered Part and the Coating

**Figure 5:**
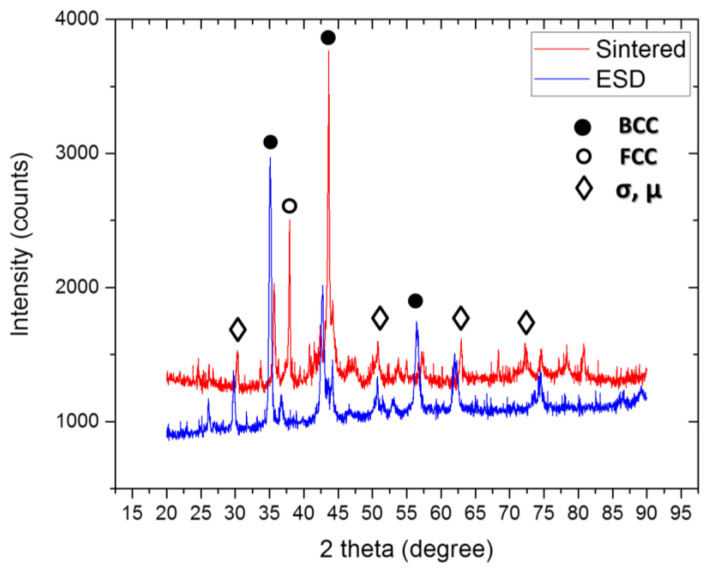
XRD pattern for the bulk and coated sample of CoCrFeNiMo0.85.

The results of the XRD analyses performed for the sintered part from which the electrode was machined and the coating are presented in Figure 5.

We can observe an increase in μ and σ phase for the coating. CoCrFeNiMo0.85 presented a VEC calculated previously [22] of 7.83. This means a mixture of FCC and BCC phases with a slightly increased amount of FCC phase. For the coating, we can observe that the FCC peak is shifted to the left probably due to the inference with the substrate. The BCC phase is present, but also there is an increased amount of σ phase rich in Co, Cr and Fe and μ phase rich in Mo.

### 3.3. Pull off Test for CoCrFeNiMo_0.85_ Coating

**Figure 6:**
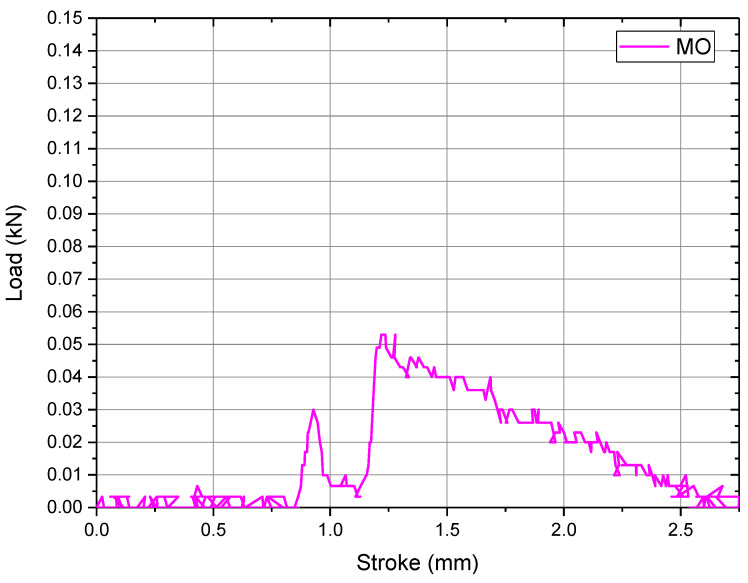
Load chart for pull off testing on CoCrFeNiMo_0.85_ high-entropy alloy coating.

For the pull off testing experiments, the results present that the maximum adhesion resistance was reached at 0.67 MPa for an area of contact of 78.5 mm^2^, due to the epoxy resin failure. The results obtained are correlated with the following graphic presented in Figure 6 where it can be observed how the loading force was applied. In order to obtain better results, the rod movement was 1 mm/min.

The load chart presents the elastic behavior and the failure point for the epoxy resin at the maximum load. It can be also observed that the adhesion between the deposited layer and the substrate has better results compared with the layer and epoxy resin adhesion, also due to the fact that no cohesive or adhesive failures occurred during the experiments.

### 3.4. Corrosion Resistance for the CoCrFeNiMo_0.85_ ESD Coating

**Figure 7:**
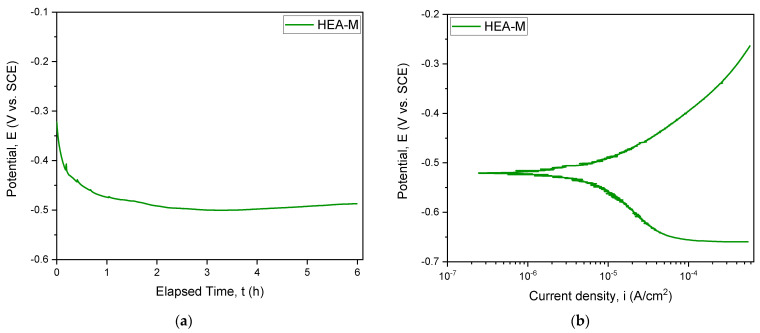
Open circuit potential evolution (**a**) and Tafel plots (**b**) for the CoCrFeNiMo_0.85_ HEA ESD coating.

A standard electrochemical experiment, potentiodynamic-polarization test, was used to study the corrosion behavior of the CoCrFeNiMo_0.85_ produced by mechanical alloying and coated using electro spark deposition technique. The variation of the potential in open circuit E_OC_ and the potentiodynamic polarization curve for the as-cast CoCrFeNiMo_0.85_ sample are shown in Figure 7.

In Table 2, the parameters for the CoCrFeNiMo_0.85_ electrochemically tested in 3.5% wt.% NaCl solution, at room temperature, is presented. The following parameters have been determined to characterize the corrosion resistance of the investigated samples: open circuit potential after 6h (E_OC_), corrosion potential (E_corr_), corrosion current density (i_corr_), cathodic slope (β_c_) and anodic slope (β_a_). With the aid of the parameters determined by Tafel extrapolation technique, the polarization resistance (R_p_) has been calculated.

The polarization resistance of the investigated alloy was also calculated based on the Stern-Geary equation (Equation (1)) [28,29]:

(1) $$R_{p}=\frac{1}{2.3}\frac{\beta_{a}|\beta_{c}|}{\beta_{a}+\left|\beta_{c}\right|}\frac{1}{\begin{array}{c} i_{corr} \end{array}}$$

where: *β_a_*—anodic slope,

*β_c_*—cathodic slope,

*i_corr_*—corrosion current density (µA/cm^2^)

The corrosion rate was calculated according to ASTM G102-89 (2004) [30] with the following equation:

(2) $$CR=K_{i}\frac{i_{corr}}{\rho }EW$$

where: *CR*—corrosion rate (mm/year)

*K_i_*—3.27 × 10^−3^

ρ—material’s density (g/cm^3^)

*i_corr_*—current density of material (µA/cm^2^) and EW–equivalent weight (g)

## 4. Discussion

The effect of adding molybdenum to the CoCrFeNi alloy [27] results in improved corrosion resistance of the high-entropy alloy in highly corrosive liquid media. Our preliminary testings were performed in situ on the bulk as-cast CoCrFeNiMo high-entropy alloy [31], in a geothermal power plant in Iceland and the corrosion rate obtained was very low, 0.00034 mm/year as mentioned. When the bulk as-cast high-entropy alloy was tested in 3.5 wt.% NaCl solution at room temperature, it revealed a low corrosion rate of 0.0072 mm/year and, when compared with the results for stainless steel [32] in the same environment, indicated that CoCrFeNiMo could represent a corrosive resistant solution that can be considered for geothermal in-work equipment [33].

An analysis of the XRD data presented in Figure 5 reveals that for the sintered part, the mixture between FCC and BCC phase is dominant. For the coated sample we can observe an increase in the phases that are rich in Co, Cr and Fe-σ phase and rich in Mo-μ phase. This phenomenon could be explained by the electro spark deposition process. The consolidated electrode (presented in XRD pattern for sintered part in Figure 5) is melted and rapidly cooled during the coating process. The transformations are induced by the temperature difference and the local solute solidification during the coating process. Thus, some of the BCC phases present in the sintered part are broadening since the σ and μ are more present in the coating.

The advantages of this electro spark deposition include the high adhesion of the resulting coatings, the possibility of local processing of large-sized parts, its relative simplicity, low energy consumption, a high environmental compatibility, and the possibility of process automation. Electro spark deposition has been successfully used to protect nickel alloys against oxidation [33,34,35]. Novel promising coatings that would be resistant to long-term high-temperature oxidation, corrosion action, and wear are currently being sought for [36]. Thus, the material was processed by mechanical alloying, consolidated and machined into an electrode suitable for the electro spark deposition technique. A stainless steel substrate was coated with CoCrFeNiMo_0.85_ high-entropy alloy and analyzed. The results indicate a compact and homogenous layer. TGood adhesion was observed in the microstructure analyses and was correlated with the pull off testing results, where no cracks or exfoliations were observed. The microstructure was presenting some splats and micro pores in the upper part as it can be seen in Figure 4b. The layer close to the substrate is homogeneous and the pores seem not to be present in that area. The good adhesion of the layer with the substrate is proved by the pull off test and also the microstructure reveals a continuous bonded layer next to the substrate.

**Figure 8:**
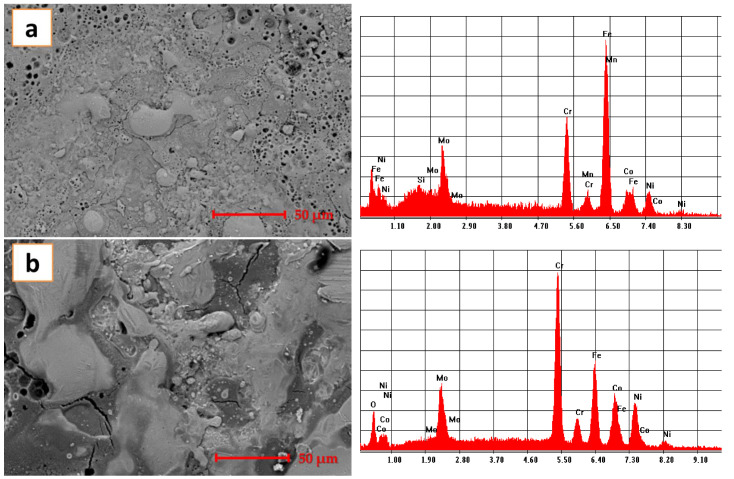
CoCrFeNiMo_0.85_ coating before (**a**) and after (**b**) exposure to corrosion.

Corrosion testing was performed in 3.5 wt.% NaCl solution and the calculated value of the corrosion rate of 0.00016 mm/year indicating that the studied material is suitable for aggressive media. The microstructures of the sample before and after corrosion are presented in Figure 8.

In Figure 8a, the microstructure presents a series of pores and splats due to the ESD technique of applying coatings. The pores are on the superficial layer. The ESD techniques resulted in multilayer coatings to achieve good protection for the substrate. Applying successive layers provides a homogeneous coating but due to the BCC structure prone to be formed for this high-entropy alloys, pores are also formed. In the second image, the pores are almost covered by the appeared passivation layer. In the results of the EDS analyses, we can also observe the oxygen component of the occurred passivation layer. Probably, part of the chromium segregated from the coating and formed the passivation layer, as we can see from the EDS analyses results.

By comparing the obtained results, the lowest corrosion rate value was obtained for the deposited coatings of CoCrFeNiMo_0.85_ high-entropy alloy produced by the solid-state processing route. Even if the Eoc has a more electronegative value than the bulk as-cast alloy [33] the alloy developed rapidly a corrosion protection film and the corrosion current intensity was very small. Probably the increased porosity of the coating in the upper part determined the more electronegative value for the open circuit potential, due to the fact that the passivation was produced in a longer period of time. The calculated corrosion rate is also very small indicating good corrosion behavior for the tested alloy. The calculated polarization resistance has a value higher than 316 L SS (22.50 kΩ × cm^2^) [37] indicating that the coating may provide good protection for the stainless steel used in the geothermal power plants.

Lyu et al. [38] determined the corrosion potential Ecorr (mV) for an alloy with 0.2 Mo, CoCrFeNiMo_0.2_. The value determined by them, in the same corrosion conditions 3.5%NaCl at room temperature is much more negative, −907 mV, indicating a higher susceptibility to corrosion. In addition, i_corr_ was determined and the value is 7.75 μA/cm^2^, confirming the hypothesis of higher corrosion susceptibility. Increasing the Mo content to 0.85 resulted in a more positive value for corrosion potential Ecorr and a much lower value for the corrosion current, i_corr_ indicating a better corrosion behavior in this case.

## 5. Conclusions

The CoCrFeNiMo_0.85_ high-entropy alloy was developed by solid-state processing. Mechanical alloying followed by pressing and sintering was employed to achieve a good compact sample that was further machined into an innovative electrode.

With the newly obtained electrode, a coating was deposited on a 316 L stainless steel substrate. The coating was homogeneous but slightly porous in the upper part. 

The pull off test and microstructure investigations revealed a good, adhesive coating and homogenous in the cross-section. The coating is also bonded by the substrate and in the lower part, closer to the substrate, where no pores or cracks are present.

The corrosion results indicate a corrosion rate of 0.00016 mm/years, very low value meaning that the coating could perform as a corrosion protection coating in an aggressive environment.

## Figures and Tables

**Table 1:** Electro spark deposition parameters for HEA coatings on stainless steel substrates.

Coating	Substrate	Gas Shield	Voltage (V)	Capacitance (µF)	Frequency (Hz)
CoCrFeNiMo_0.85_	SS 316 L	Argon	100	20	200

**Table 2:** CoCrFeNiMo_0.85_ alloy parameters for electrochemical testing in chloride-containing solution at room temperature with 3.5 wt.% NaCl.

Sample	E_oc_ (mV)	E_cor_ (mV)	i_corr_ (μA/cm^2^)	β_c_ (mV)	β_a_ (mV)	Rp (kΩ × cm^2^)	CR (mm/Year)
HEA-M	−487	−520	0.0011	222.53	140.09	33.980	0.00016

## Data Availability

Not applicable.
